# Investigation of the Telomere Length in PERI‐Implant Oral Mucosa Cells

**DOI:** 10.1111/jop.70061

**Published:** 2025-09-11

**Authors:** Fernando Costa Giffoni, Thaís Ellen Chaves Gomes, Priscila Nazaré de Souza, Paula Valente e Silva, Luiz Alexandre Viana Magno, Amanda Lage Cândido, Marcelo Faria Lasmar, Sebastião Guilherme de Oliveira Neto, Marcus Vinicius Lucas Ferreira, Thiago Moreira Gonçalves Araujo, Luara Isabela dos Santos, Bruno Ramos Chrcanovic, Ricardo Santiago Gomez, Roberta Rayra Martins‐Chaves

**Affiliations:** ^1^ Faculty of Medical Sciences of Minas Gerais Belo Horizonte Brazil; ^2^ School of Dentistry, Federal University of Minas Gerais Belo Horizonte Brazil; ^3^ Molecular Studies Core in Oncology (Núcleo de Estudos Moleculares em Oncologia ‐ NEMO) Faculty of Medical Sciences of Minas Gerais (FCMMG) Belo Horizonte Minas Gerais Brazil; ^4^ Department of Oral and Maxillofacial Surgery and Oral Medicine, Faculty of Odontology Malmö University Malmö Sweden

**Keywords:** biocompatibility, cellular senescence, dental implants, peri‐implant mucosa, telomere length

## Abstract

**Background:**

The biological effects of dental implants on peri‐implant tissues have been widely investigated. Recent reports of oral squamous cell carcinoma (OSCC) cases adjacent to dental implants have raised concerns regarding the potential impact of implant materials on cellular aging and oncogenic transformation. Telomeres, which protect chromosome ends, undergo progressive shortening and play a critical role in cellular senescence and tumorigenesis. However, the impact of dental implants on telomere length in peri‐implant mucosa remains unclear.

**Objective:**

This study aimed to compare telomere length in mucosa adjacent to dental implants with that of gingival tissue associated with healthy teeth.

**Methods:**

A paired cross‐sectional study was conducted with 16 patients who had dental implants for at least 1 year. Swabs were collected from the peri‐implant mucosa and healthy gingival mucosa of the same patient. Telomere length was assessed using quantitative PCR, with the relative telomere‐to‐single‐copy‐gene ratio (T/C) calculated using the 2^−∆∆Cq^ method.

**Results:**

Telomere length in the peri‐implant mucosa was not significantly different from that in the healthy gingival mucosa (*p* = 0.117).

**Conclusion:**

These findings suggest that dental implants do not alter telomere length in adjacent mucosal cells.

## 1 Introduction

The biological effects of implants on the surrounding hard and soft tissues have been a topic of investigation for decades [1, 2]. The study of its interface with those tissues is important not only because of the desired long‐term osseointegration of the implant, but also for the importance of understanding how cells react to dental implant materials.

In the last years, an increased number of studies have reported oral squamous cell carcinoma (OSCC) cases around dental implants. Most patients with OSCC around dental implants were women without known risk factors, and the lesions may present clinical and radiographic features that could resemble peri‐implantitis [3]. Although some systematic reviews of OSCC and oral potentially malignant disorders (OPMD) around implants have not established a clear association between dental implants and oral cancer [3], the possibility cannot be ruled out without more robust causal evidence.

If dental implants have the potential to release carcinogenic compounds, different study designs are necessary to clarify their effects in biological tissues. Telomeres are cap‐like DNA protein structures that protect chromosome ends. They undergo a progressive shortening with cell division, but when they reach a critical limit, cells undergo replicative senescence. Telomere dysfunction has been associated with oncogenic transformation [4]. Furthermore, different studies over the years have demonstrated shortened telomeres in OPMD [5]. Taken all these data together, we raised the hypothesis of whether dental implants could cause telomere length dysfunction of the oral adjacent mucosa cells. For this purpose, we compared the telomere length in paired swabs from the oral mucosa adjacent to dental implants and healthy gingiva from the same patient.

Informed consent was obtained from all subjects, and this study was approved by the Ethics Committee of the Faculdade de Ciências Médicas de Minas Gerais, Belo Horizonte, Brazil. Scrapings from the gingival mucosa of healthy teeth and peri‐implant mucosa without inflammation from 16 patients were collected, and the genomic DNA was extracted using the QIAamp DNA Blood Mini Kit (QIAGEN, Hilden, Germany), following the manufacturer's protocol. Relative telomere length was quantified by qPCR using the Cawthon method [6], which determines the ratio of telomeric repeat copy number (T) to a single‐copy reference gene (C), 36B4. Each reaction used 75 ng of DNA and was performed in triplicate in 96‐well plates, using the Invitrogen Platinum Taq Kit and an ABI‐7500 machine. Primer pairs were specific for telomeric repeats and the 36B4 gene. SYBR‐Green was used as the fluorescent reporter for amplicon detection. Cycle threshold (Ct) values were averaged across technical replicates. Samples with a standard deviation (SD) > 0.25 among replicates were excluded. The relative T/C ratio between samples was calculated using the 2^−ΔΔCt^ method.

**FIGURE 1:**
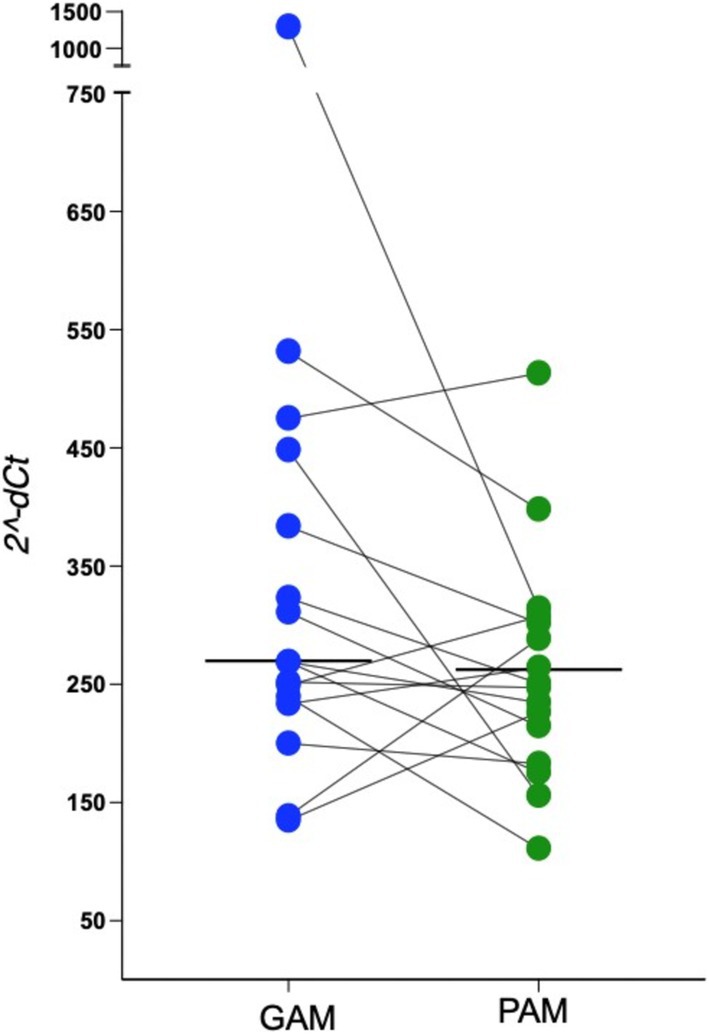
Paired comparison of relative telomere length (2^−ΔCt^) between gingival mucosa associated with natural teeth (GAM) and peri‐implant mucosa (PAM) from the same individuals (*n* = 16). Each pair of dots represents the two tissue types from the same patient, connected by a line. Horizontal lines represent group medians.

The ∆Cq and 2^−dCt^ values of the health gingival mucosa (GAM) and peri‐implant mucosa (PAM) are shown in Figure 1. The 2^−∆Cq^ represents the relative expression of each sample, based on the comparison between the telomere and the control gene. As the data did not follow a normal distribution, the Wilcoxon signed‐rank test was used for comparative analysis. The 2^−∆Cq^ median of the GAM group was 268.9 (IQR: 197.0), while for the PAM group it was 249.0 (IQR: 115.1), with no statistically significant difference between them (*p* = 0.117) (Figure 1). Although not statistically significant, there was a tendency toward higher 2^−ΔCt^ values in the GAM group, which may justify further investigation with a larger sample size or improve control of potential confounding variables. Although tobacco and alcohol are the main risk factors for oral cancer, some studies have suggested that metallic dental materials, such as dental implants, can leach ions with mutagenic potential into the oral mucosa [7]. On the other hand, no genotoxicity of titanium alloys on oral mucosa tissues was observed when assessed by the micronucleus assay [8].

Studies have shown that critically short telomeres are associated with cancer initiation and progression [9]. Therefore, information about possible changes in telomere length in mucosa associated with implants is crucial for gaining a better understanding of its biocompatibility. This study was designed as a paired analysis, using each patient's healthy gingival mucosa as a control to account for individual genetic and environmental variability, which may influence telomere length.

Our results did not reveal significant differences in the telomere length between peri‐implant mucosa and healthy gingival mucosa. This suggests that dental implants do not promote telomere shortening in adjacent oral tissues. However, this does not exclude the possibility that metals used in implants may lead to other biological alterations. For example, it has been reported that titanium particles released from the implant surface may activate DNA damage response pathways (DDR) in oral epithelium [10]. Nonetheless, our findings support the favorable biocompatibility of titanium implants. On the other hand, it is important to highlight that oral potentially malignant and neoplastic lesions do not exhibit only changes in telomere length. Other molecular alterations, such as aneuploidy or loss of heterozygosity, are also found in these lesions. Therefore, new molecular studies should be encouraged to better understand the effects of implants on oral mucosal health.

In conclusion, our study shows that dental implants do not cause changes in telomere length. Further research with large cohorts and expanded molecular profiling is necessary to validate these findings and explore other potential biological effects of dental implants.

## Author Contributions

Conceptualization: R.R.M.‐C., R.S.G. B.R.C. Investigation: F.C.G., T.E.C.G., R.R.M.‐C., and R.S.G. Methodology: R.R.M.‐C., T.E.C.G, L.A.V.M., and F.C.G. Sample collection: F.C.G., P.N.S., P.V.S., M.F.L., S.G.O.N., M.V.L.F., and T.M.G.A. Writing – original draft: R.S.G., R.R.M.‐C., B.R.C. Writing – review: F.C.G., R.R.M.‐C., P.N.S., P.V.S., M.F.L., S.G.O.N., M.V.L.F., T.M.G.A., T.E.C.G., L.A.V.M., R.R.M.C., B.R.C. and R.S.G. Editing: R.R.M.C., R.S.G., and B.R.C. Formal analysis: R.R.M.‐C. Project administration: R.S.G. and R.R.M.‐C. All authors have read and agreed to the published version of the manuscript.

## Ethics Statement

This study was approved by the Research Ethics Committee of the Faculty of Medical Sciences of Minas Gerais (CAAE:75218323.4.0000.5134).

## Consent

All participants provided written informed consent by signing the Informed Consent Form.

## Conflicts of Interest

The authors declare no conflicts of interest.

## Data Availability

The data that support the findings of this study are available on request from the corresponding author. The data are not publicly available due to privacy or ethical restrictions.
